# Efficacy of Xpert in tuberculosis diagnosis based on various specimens: a systematic review and meta-analysis

**DOI:** 10.3389/fcimb.2023.1149741

**Published:** 2023-05-02

**Authors:** Xue Gong, Yunru He, Kaiyu Zhou, Yimin Hua, Yifei Li

**Affiliations:** Department of Pediatrics, Ministry of Education Key Laboratory of Women and Children’s Diseases and Birth Defects, West China Second University Hospital, Sichuan University, Chengdu, Sichuan, China

**Keywords:** Xpert, tuberculosis, specimens, meta-analysis, systematic review

## Abstract

**Objective:**

The GeneXpert MTB/RIF assay (Xpert) is a diagnostic tool that has been shown to significantly improve the accuracy of tuberculosis (TB) detection in clinical settings, with advanced sensitivity and specificity. Early detection of TB can be challenging, but Xpert has improved the efficacy of the diagnostic process. Nevertheless, the accuracy of Xpert varies according to different diagnostic specimens and TB infection sites. Therefore, the selection of adequate specimens is critical when using Xpert to identify suspected TB. As such, we have conducted a meta-analysis to evaluate the effectiveness of Xpert for diagnosis of different TB types using several specimens.

**Methods:**

We conducted a comprehensive search of several electronic databases, including PubMed, Embase, the Cochrane Central Register of Controlled Trials, and the World Health Organization clinical trials registry center, covering studies published from Jan 2008 to July 2022. Data were extracted using an adapted version of the Checklist for Critical Appraisal and Data Extraction for Systematic Reviews of Prediction Modeling Studies. Where appropriate, meta-analysis was performed using random-effects models. The risk of bias and level of evidence was assessed using the Quality in Prognosis Studies tool and a modified version of the Grading of Recommendations Assessment, Development, and Evaluation. RStudio was utilized to analyze the results, employing the *meta4diag*, *robvis*, and *metafor* packages.

**Results:**

After excluding duplicates, a total of 2163 studies were identified, and ultimately, 144 studies from 107 articles were included in the meta-analysis based on predetermined inclusion and exclusion criteria. Sensitivity, specificity and diagnostic accuracy were estimated for various specimens and TB types. In the case of pulmonary TB, Xpert using sputum (0.95 95%CI 0.91–0.98) and gastric juice (0.94 95%CI 0.84–0.99) demonstrated similarly high sensitivity, surpassing other specimen types. Additionally, Xpert exhibited high specificity for detecting TB across all specimen types. For bone and joint TB, Xpert, based on both biopsy and joint fluid specimens, demonstrated high accuracy in TB detection. Furthermore, Xpert effectively detected unclassified extrapulmonary TB and tuberculosis lymphadenitis. However, the Xpert accuracy was not satisfactory to distinguish TB meningitis, tuberculous pleuritis and unclassified TB.

**Conclusions:**

Xpert has exhibited satisfactory diagnostic accuracy for most TB infections, but the efficacy of detection may vary depending on the specimens analyzed. Therefore, selecting appropriate specimens for Xpert analysis is essential, as using inadequate specimens can reduce the ability to distinguish TB.

**Systematic review registration:**

https://www.crd.york.ac.uk/prospero/display_record.php?RecordID=370111, identifier CRD42022370111.

## Introduction

Tuberculosis (TB) infection, caused by Mycobacterium tuberculosis, is a leading cause of mortality worldwide and ranks among the deadliest infectious diseases, including HIV and malaria. Despite global efforts, TB remains a significant public health threat, particularly in developing and underdeveloped countries. The World Health Organization (WHO) has reported that an estimated 10.4 million people contract new TB infections yearly, with 1.8 million TB-related deaths occurring annually (Holmes et al., 2017; Harding, 2020). Furthermore, pediatric patients are particularly vulnerable, with an annual mortality rate of 0.2 million. However, the features of *M. tuberculosis* make it challenging to establish a definitive diagnosis in a timely manner, resulting in over 40% of patients not receiving a prompt diagnosis. Moreover, latent TB infection can lead to substantial morbidity and mortality, with negative socioeconomic consequences (Vitoria et al., 2009). Therefore, improved TB diagnostic procedures and techniques are urgently needed.

Accurate detection of *M. tuberculosis* is crucial for diagnosing TB, a respiratory-transmitted disease that primarily affects the lungs. Additionally, TB infection can result in tissue damage, particularly in young children and adolescents, who have a higher incidence of TB infection than adults (Holmes et al., 2017; Lamb and Starke, 2017). Due to the nonspecific symptoms and the paucibacillary nature of the disease, TB diagnosis is challenging. Diagnostic approaches for TB rely on clinical signs and symptoms. Non-sputum-based tests, such as tuberculin skin tests and chest radiography, have insufficient sensitivity and specificity (Khan and Starke, 1995).

The GeneXpert MTB/RIF assay (Xpert) was the first point-of-care assay for TB, and was endorsed by the WHO in 2010 (WHO Guidelines Approved by the Guidelines Review Committee, 2011). Xpert has demonstrated improved efficacy in detecting intrapulmonary and extrapulmonary TB. However, the sensitivity of Xpert has been found to vary depending on the specimens used for analysis, namely sputum, stool, bronchoalveolar lavage fluid (BALF), gastric juice, interstitial fluid, and biopsies. Xpert’s estimated specificity among all specimens is generally higher than 98% (Kay et al., 2020). Moreover, Xpert has improved the efficacy of TB diagnostic procedures, particularly in the early phase of infection. Nevertheless, the accuracy rate of Xpert varies between specimens and specific types of TB. Therefore, selecting optimal specimens is critical for applying Xpert in identifying specific suspected TB infections (Howard-Jones and Marais, 2020; Gaensbauer, 2021; Gebre et al., 2021; Kabir et al., 2021; Maharjan et al., 2021). Accordingly, we conducted a meta-analysis to evaluate the efficacy of Xpert in detecting different types of TB in different samples, aiming to provide evidence of the best specimens for each type of TB infection.

## Materials and methods

### Study protocol

This metanalysis was conducted by following a predetermined protocol in accordance with the recommendations of a guideline for systematic reviews of diagnostic studies. Data collection and reporting followed the Preferred Reporting Items for Systematic Reviews and Meta-Analyses (PRISMA) Statement (PROSPERO; CRD42022370111).

### Search strategy

A comprehensive search strategy was employed to identify relevant publications, utilizing multiple databases, including PubMed, Embase, the Cochrane Central Register of Controlled Trials, and the World Health Organization clinical trials registry center. The search strategy included various Medical Subject Headings (MeSH) terms and keywords related to tuberculosis and Xpert assay. Specifically, the search terms used were “tuberculosis”[All Fields] OR “tuberculosis”[MeSH Terms] OR “tuberculosis”[All Fields] OR “tuberculoses”[All Fields] OR “tuberculosis s”[All Fields] OR (“tuberculosis”[MeSH Terms] OR “tuberculosis”[All Fields] OR (“tuberculosis”[All Fields] AND “infection”[All Fields]) OR “tuberculosis infection”[All Fields] OR “latent tuberculosis”[MeSH Terms] OR (“latent”[All Fields] AND “tuberculosis”[All Fields]) OR “latent tuberculosis”[All Fields] OR (“tuberculosis”[All Fields] AND “infection”[All Fields])) AND (“Xpert”[All Fields] OR (“GeneXpert”[All Fields] AND “mtb rif”[All Fields])). As the technology of Xpert had been introduced in clinical since 2009, the search included publications from Jan 2008 to July 2022.

### Study selection

The citations retrieved from the systematic search were initially screened based on their titles and/or abstracts. Relevant reports were then retrieved as complete manuscripts and evaluated for compliance with the inclusion and exclusion criteria. The following inclusion criteria were used: 1) Xpert examination was performed on any type of specimen in all individuals; 2) the study involved a diagnostic test or performance design; 3) a definitive diagnostic gold standard was provided for TB infection diagnosis in all the studies; 4) the data to obtain the results of true positive (TP), false positive (FP), false negative (FN), and true negative (TN) of Xpert examination were available. Alternatively, sensitivity, specificity, and actual sample size data could be provided, which could be calculated or converted into TP, FP, FN, and TN. The following exclusion criteria were used: 1) inclusion of the same cohort that had been studied in another study, with only the newest cohort data being included; 2) essential data for pooled analysis or quality assessment were not retrievable; 3) absence of a gold standard setup; and 4) exclusion of case reports or conference articles.

### Data collection and assessment of study quality

Initially, we manually searched the reference lists of all included studies, previous systematic reviews, and articles citing the included studies using Google Scholar. Subsequently, relevant reports were obtained as full-text manuscripts and examined for adherence to the inclusion and exclusion criteria. To assess the methodological quality of each study, we employed the 14-item Quality Assessment of Diagnostic Accuracy Studies (QUADAS) checklist. Each item was assessed with a response of “yes,” “no,” or “unclear” while evaluating the included studies. Because the quality assessment is intricately linked to reporting results, a well-designed study could have received a poor score if the methods and results were not reported in sufficient detail. Therefore, we presented the assessment in a descriptive format rather than as a numerical score.

### Publication bias

The possibility of publication bias was examined using funnel plots and the Risk-of-bias VISualization (*Robvis*) tool in the R programming environment (version 4.2.0). The presence of an asymmetric distribution of data points in the funnel plot and the quantified results obtained from the traffic light plot was used to determine any risk of bias. This plot displays each risk-of-bias judgment in a matrix, with domains along the horizontal and results/studies down the vertical axis, similar to the data set, and a weighted bar plot, which depicts the proportion of information with each risk-of-bias judgment separately for each domain in the specified assessment tool (McGuinness and Higgins, 2021).

### Sensitivity analyses

A sensitivity analysis was performed by systematically excluding one set of study data at a time to investigate whether any individual study was exerting an excessive influence on the overall analysis. The pooled results were then reassessed to determine whether there was a significant alteration in the findings. These analyses were conducted for each study included in this work.

### Statistical analyses

The data were analyzed using R and RStudio software version 4.2.0. The diagnostic efficacy of different specimens of Xpert in distinguishing various types of TB was measured using sensitivity, specificity, diagnostic odds ratio (DOR), and summary receiver operating characteristics (SROC) curve. Sensitivity was defined as the proportion of patients diagnosed with TB by the gold standard correctly identified by the positive results of different specimens using Xpert. Specificity was defined as the non-TB cases correctly identified by the negative results of different samples using Xpert. The crosshair plot for the Xpert data set was generated by displaying the posterior means for each study as the summary points, along with paired lines showing the corresponding 95% confidential intervals (95% CI) for sensitivity and false positive rate (1-specificity). The confidence intervals presented in particular results were related to the confidence level, sample size, and associated factors. The crosshair plot displayed the individual studies in receiver operating characteristic (ROC) space with paired confidence intervals representing sensitivity and specificity (Phillips et al., 2010). The SROC was also plotted based on the sensitivity and specificity combination.

The area under the curve (AUC) value was calculated as a global measurement of diagnostic accuracy performance. Posterior density plots were also generated to estimate the correlation between the two linear predictors. The posterior density plot showed the relative importance of the two-study metrics. The risk of bias was assessed using the *Robvis* and *metafor* packages in RStudio. Pooled sensitivity and specificity, based on various specimens in TB detection using Xpert, were measured using the meta4diag package in RStudio. Moreover, one set of study data was systematically removed to determine whether any single study was incurring undue weight in the analysis. The pooled results for the remaining studies were reanalyzed to determine whether the results had a significant change. Sensitivity analysis was conducted for every study.

## Results

### Study evaluation

A total of 2163 studies were initially identified through literature search and abstracts. Following the application of inclusion and exclusion criteria, 1908 citations were excluded, leaving 207 articles for careful evaluation. Ultimately, 101 articles were excluded, 58 of which did not provide necessary data on TP, FP, FN, and TN. Further, 37 studies did not report the gold standard utilized, 5 studies were designed for TB drug resistance without a diagnostic test design, and 1 article was a case report (**Supplementary Figure 1**). The essential information and basic characteristics of the 144 included studies from 107 articles (Armand et al., 2011; Causse et al., 2011; Friedrich et al., 2011; Ligthelm et al., 2011; Malbruny et al., 2011; Vadwai et al., 2011; Moure et al., 2012; Tortoli et al., 2012; Bates et al., 2013; Christopher et al., 2013; Porcel et al., 2013; Van Rie et al., 2013; Weyer et al., 2013; Zmak et al., 2013; Ablanedo-Terrazas et al., 2014; Biadglegne et al., 2014; Coetzee et al., 2014; Javed et al., 2014; Litao et al., 2014; Lusiba et al., 2014; Meldau et al., 2014; Pang et al., 2014; Scott et al., 2014; Theron et al., 2014; Trajman et al., 2014; Coleman et al., 2015; Gu et al., 2015; Kim et al., 2015; Liu et al., 2015; Rufai et al., 2015; Singh et al., 2015; Solomons et al., 2015; Agrawal et al., 2016; Bajrami et al., 2016; Bholla et al., 2016; Chew et al., 2016; Dhooria et al., 2016; Held et al., 2016; Jo et al., 2016; Marcy et al., 2016; Mazzola et al., 2016; Reechaipichitkul et al., 2016; Sauzullo et al., 2016; Arockiaraj et al., 2017; Aslam et al., 2017; Che et al., 2017; Chikaonda et al., 2017; Hasan et al., 2017; Jin et al., 2017; Kawkitinarong et al., 2017; Lu et al., 2017; Massi et al., 2017; Meyer et al., 2017; Rufai et al., 2017; Tang et al., 2017; Tang et al., 2017; Ullah et al., 2017; Walters et al., 2017; Yang et al., 2017; Yu et al., 2017; Bahr et al., 2018; Castro et al., 2018; Christopher et al., 2018; Cresswell et al., 2018; Fan et al., 2018; Kasa Tom et al., 2018; Khan et al., 2018; Li et al., 2018; Lu et al., 2018; Myo et al., 2018; Pereira et al., 2018; Perez-Risco et al., 2018; Rahman et al., 2018; Samuel et al., 2018; Sharma et al., 2018; Akhter et al., 2019; Allahyartorkaman et al., 2019; Creswell et al., 2019; Dahale et al., 2019; Fakey Khan et al., 2019; Galal El-Din et al., 2019; Peize et al., 2019; Piersimoni et al., 2019; Rasheed et al., 2019; Sun et al., 2019; Tadesse et al., 2019; Talib et al., 2019; Wang et al., 2019; Wu et al., 2019; Donovan et al., 2020; Phetsuksiri et al., 2020; Shao et al., 2020; Sharma et al., 2020; Solanki et al., 2020; Ssengooba et al., 2020; Sun et al., 2020; Tiamiyu et al., 2020; Wang et al., 2020; Xia et al., 2020; Yeong et al., 2020; Yu et al., 2020; Zhou et al., 2020b; Zhou et al., 2020a; Kohli et al., 2021; Liu et al., 2021; Parigi et al., 2021; Pierre-Louis et al., 2021) are summarized in **Supplementary Table 1**.

### Study quality

Quality assessment of diagnostic accuracy studies was conducted using the QUADAS list. Most of the included studies received satisfactory scores on the essential items in the QUADAS list. However, several studies lacked complete reporting on certain items, such as uninterpretable test results and excluded cases (**Supplementary Figure 2** and **Supplementary Table 2**).

### Risk of bias

**Figure 1:**
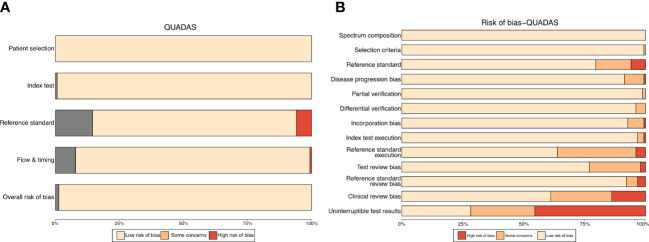
Traffic light plot **(A)** for QUADAS-s scores of included studies. Weighted bar plot **(B)** shows the proportion of information with each risk-of-bias judgement separately for each domain in the assessment.

Assessment of the risk of bias was visualized using the *robvis* (Risk-of-bias VISualization) package (McGuinness and Higgins, 2021). The traffic light plot presents every risk-of-bias judgment in a matrix, while the weighted bar plot displays the proportion of information for each risk-of-bias judgment separately for each domain in the assessment (**Figures 1A, B**). To evaluate the publication bias of included studies, funnel plots were utilized, with each dot representing a study and the distance between each dot and the horizontal line indicating the bias of each study. The absence of any asymmetric distribution suggested no publication bias, whereas the presence of an asymmetric distribution was indicative of publication bias.

### Efficacy of Xpert in identifying pulmonary TB on various specimens

**Figure 2:**
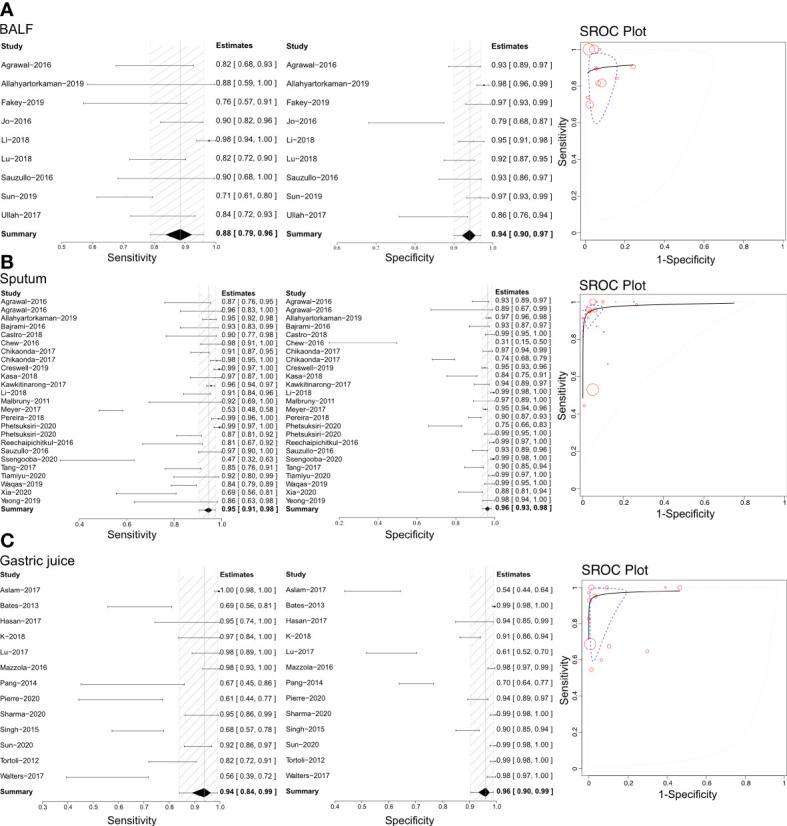
The sensitivity, specificity and SROC curve of different samples for diagnosing pulmonary TB. **(A)** The diagnostic efficiency on BALF sample based Xpert analysis; **(B)** The diagnostic efficiency on sputum sample based Xpert analysis; **(C)** The diagnostic efficiency on gastric juice sample based Xpert analysis.

Pulmonary TB was the predominant type of TB infection. The Xpert assay was mainly used for pulmonary TB detection, and several types of specimens were evaluated to determine their accuracy. This analysis examined bronchoalveolar lavage fluid (BALF), biopsy samples, gastric juice, sputum, and stool using the Xpert assay to differentiate pulmonary TB (**Figure 2** and **Table 1**). Sensitivities, specificities, and SROCs were calculated for each analysis. Nine articles were identified for calculating the diagnostic efficacy of BALF in Xpert assay, with a sensitivity of 0.88 (95% CI 0.79–0.96) and a specificity of 0.94 (95% CI 0.90–0.97). The AUC of BALF in the Xpert assay was 0.879 ± 0.098, with an estimated AUC of 0.924 (**Figure 2A**). For sputum, 25 articles were eligible for diagnostic efficacy calculation, resulting in a sensitivity of 0.95 (95% CI 0.91–0.98) and a specificity of 0.96 (95%CI 0.93–0.98).

**Table 1:** Summary of pooled results on pulmonary TB diagnosis.

Sample for Xpet	Included researches	Sensitivity (95%CI)	Specificity (95%CI)	AUC (mean ± SD)	Publication bias
BALF	9	0.88 (0.79, 0.96)	0.94 (0.90, 0.97)	0.879 ± 0.098	Potentially exist
Sputum	25	0.95 (0.91, 0.98)	0.96 (0.93, 0.98)	0.981 ± 0.011	Absent
Gastric juice	13	0.94 (0.84, 0.98)	0.96 (0.93, 0.98)	0.953 ± 0.057	Potentially exist
Stool	2	0.79 (0.35, 0.98)	0.98 (0.93, 1.00)	0.607 ± 0.310	Absent
Biopsy	2	0.77 (0.57, 0.98)	0.86 (0.67, 1.00)	0.492 ± 0.431	Potentially exist

BALF, bronchoalveolar lavage fluid; CI, confidential intervals; SD, standard difference.

The AUC of sputum in the Xpert assay was 0.981 ± 0.011, with an estimated AUC of 0.983 (**Figure 2B**). Thirteen articles were eligible to calculate the diagnostic efficacy of gastric juice in the Xpert assay, with a sensitivity of 0.94 (95% CI 0.84–0.98) and a specificity of 0.96 (95% CI 0.93–0.98). The AUC of gastric juice using the Xpert assay was 0.953 ± 0.057, with an estimated AUC of 0.979 (**Figure 2C**). Two articles were eligible to calculate the diagnostic efficacy of stool samples using the Xpert assay, with a sensitivity of 0.79 (95%CI 0.35–0.98) and a specificity of 0.98 (95%CI 0.93–1.00). The AUC of stool in the Xpert assay was 0.607 ± 0.310, with an estimated AUC of 0.675 (**Table 1**). Two articles were identified to calculate the diagnostic efficacy of biopsy samples from bronchoscopies in the Xpert assay, with a sensitivity of 0.77 (95% CI 0.57–0.98), but the specificity failed to reach a positive calculated result. The AUC of the biopsy samples taken from a bronchoscopy in the Xpert assay was 0.492 ± 0.431, with an estimated AUC of 0.672 (**Table 1**). Crosshair and posterior density plots are presented in **Supplementary Figures 3**, **4**, respectively.

### Efficacy of Xpert in identifying bone and joint TB on various specimens

**Figure 3:**
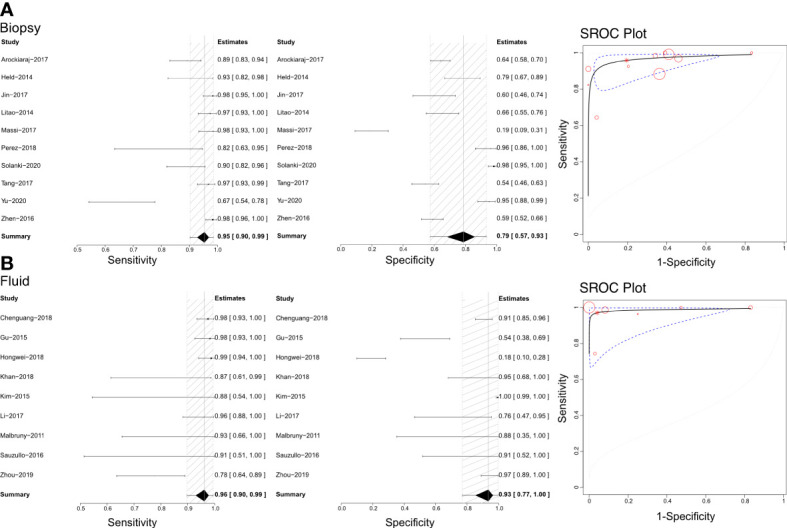
The sensitivity, specificity and SROC curve of different samples for diagnosing bone and joint TB. **(A)** The diagnostic efficiency on biopsy sample based Xpert analysis; **(B)** The diagnostic efficiency on joint fluid sample based Xpert analysis.

Based on our literature review, Xpert has been used to distinguish bone and joint TB using tissue biopsy samples and joint fluid (**Figure 3**). Therefore, we calculated each analysis’ sensitivities, specificities, and SROCs. Ten articles were eligible for use in calculating the diagnostic efficacy of tissue biopsy samples using Xpert assessment, with a sensitivity of 0.95 (95% CI 0.90–0.99) and specificity of 0.79 (95% CI 0.57–0.93). The AUC of tissue biopsy samples in the Xpert application was 0.959 ± 0.026, with an estimated AUC of 0.970 (**Figure 3A**). Additionally, nine articles were eligible to calculate the diagnostic efficacy of joint fluid using Xpert assessment, with a sensitivity of 0.96 (95% CI 0.90–0.99) and a specificity of 0.93 (95% CI 0.77–1.00). The AUC of joint fluid in the Xpert application was 0.979 ± 0.030, with an estimated AUC of 0.988 (**Figure 3B**). **Supplementary Figures 5**, **6** show the Crosshair and posterior density plots for these analyses, respectively.

### Efficacy of Xpert in identifying tuberculous lymphadenitis

**Figure 4:**
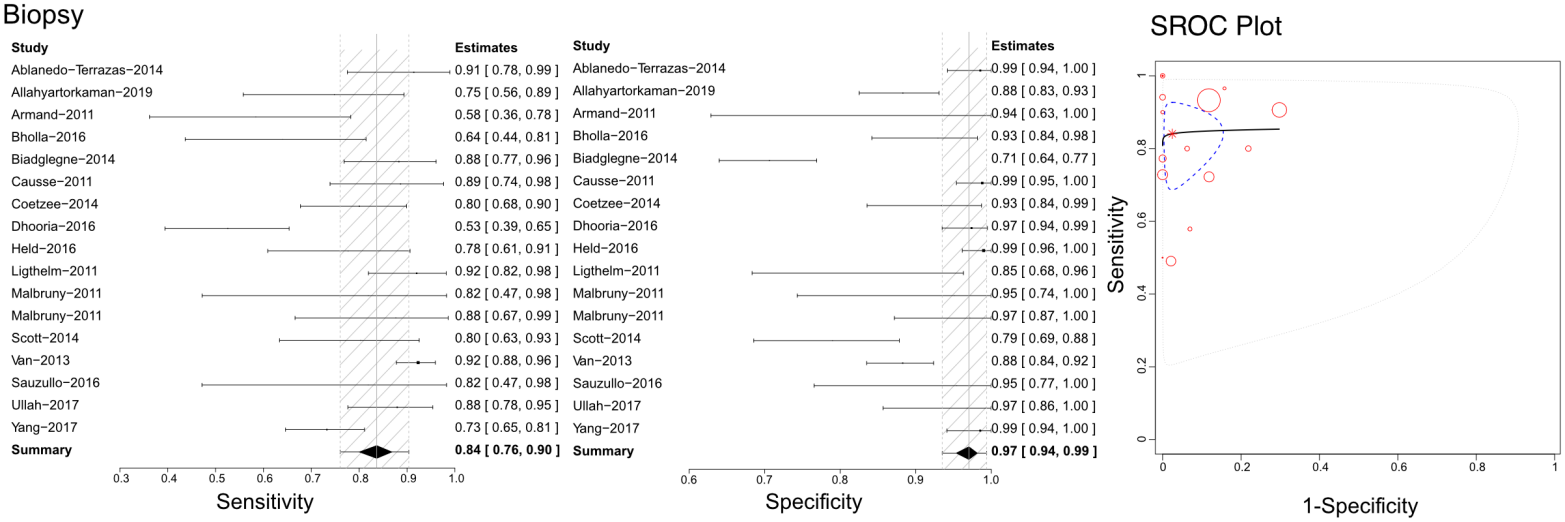
The sensitivity, specificity and SROC curve of different samples for diagnosing tuberculous lymphadenitis on biopsy sample based Xpert analysis.

Based on the literature we retrieved, Xpert has been used with lymph node biopsy samples to differentiate tuberculous lymphadenitis (**Figure 4**). Through our analysis, we calculated sensitivities, specificities, and SROCs. We identified 17 articles eligible for calculating the diagnostic efficacy of lymph node biopsy samples using Xpert assessment. The sensitivity of lymph node biopsy samples in the Xpert assessment for identifying tuberculous lymphadenitis was 0.84 (95%CI 0.76–0.90), with a specificity of 0.97 (95% CI 0.94–0.99). The AUC of tissue biopsy samples in the Xpert application was 0.753 ± 0.256, and the estimated AUC was 0.857 (**Figure 4**). Additionally, we found only one article that demonstrated the diagnostic efficacy of lymphoglandula fluid in the Xpert assay, with a sensitivity of 0.89 and a specificity of 0.91. **Supplementary Figures 7A**, **8A** present the Crosshair and posterior density plots, respectively.

### Efficacy of Xpert in identifying tuberculous meningitis

**Figure 5:**
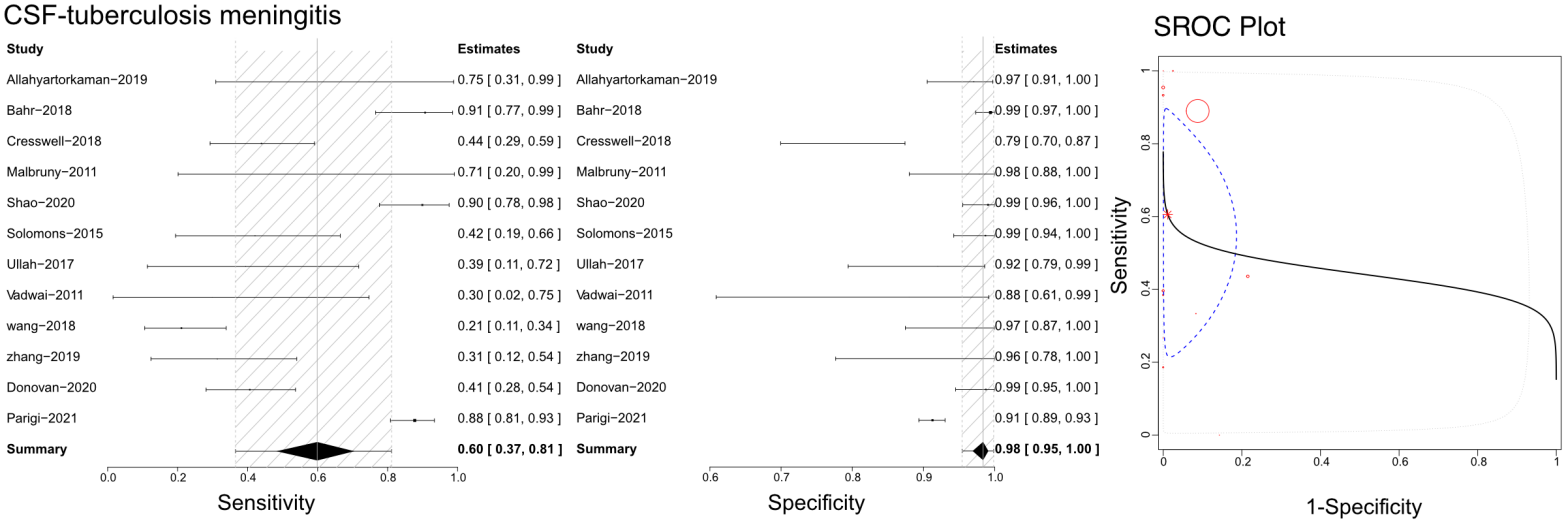
The sensitivity, specificity and SROC curve of different samples for diagnosing tuberculosis meningitis on cerebrospinal fluid sample based Xpert analysis.

Based on the literature we gathered, Xpert has been utilized with cerebrospinal fluid (CSF) to differentiate tuberculous meningitis (**Figure 5**). The sensitivities, specificities, and SROCs were computed among the assessments conducted. We identified 12 studies that qualified for evaluating the diagnostic performance of CSF in Xpert analysis. The sensitivity of CSF in Xpert evaluation for detecting tuberculous meningitis was 0.60 (95% CI 0.37–0.81), while its specificity was 0.98 (95% CI 0.95–1.00). Furthermore, the AUC of CSF in the Xpert application was 0.433 ± 0.290, and the estimated AUC was 0.444 (**Figure 5**). We present the Crosshair and posterior density plots in **Supplementary Figures 7B**, **8B**, respectively.

### Efficacy of Xpert in identifying pleural TB

**Figure 6:**
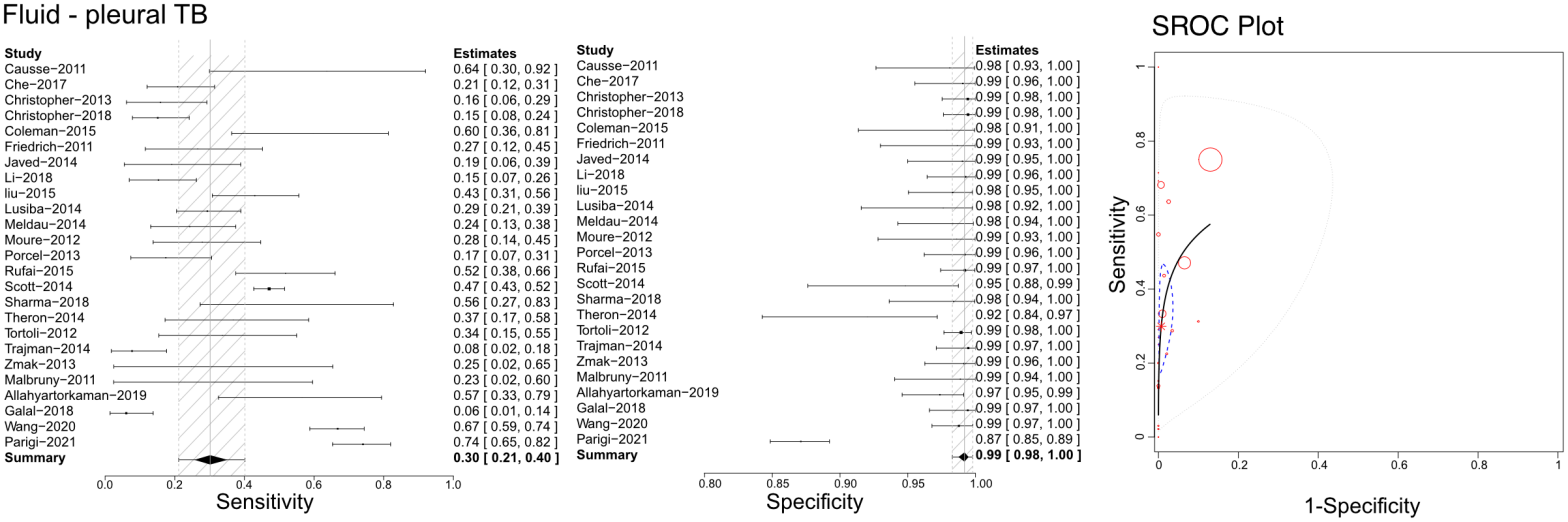
The sensitivity, specificity and SROC curve of different samples for diagnosing pleural TB on fluid sample based Xpert analysis.

Based on the literature we retrieved, Xpert has been applied with pleural fluid to differentiate pleural TB (**Figure 6**). Our analyses calculated sensitivities, specificities, and SROCs. Twenty-five articles were deemed eligible to compute the diagnostic efficacy of pleural fluid in Xpert assessment. The sensitivity of pleural fluid in Xpert assessment to detect pleural TB was 0.30 (95% CI 0.21–0.40), while its specificity was 0.99 (95% CI 0.94–1.00). Additionally, the AUC of tissue biopsy samples in the Xpert application was 0.776 ± 0.205, with an estimated AUC of 0.718 (**Figure 6**). **Supplementary Figures 7C**, **8C** present the Crosshair and posterior density plots, respectively.

### Efficacy of Xpert in identifying unclassified extrapulmonary TB

**Figure 7:**
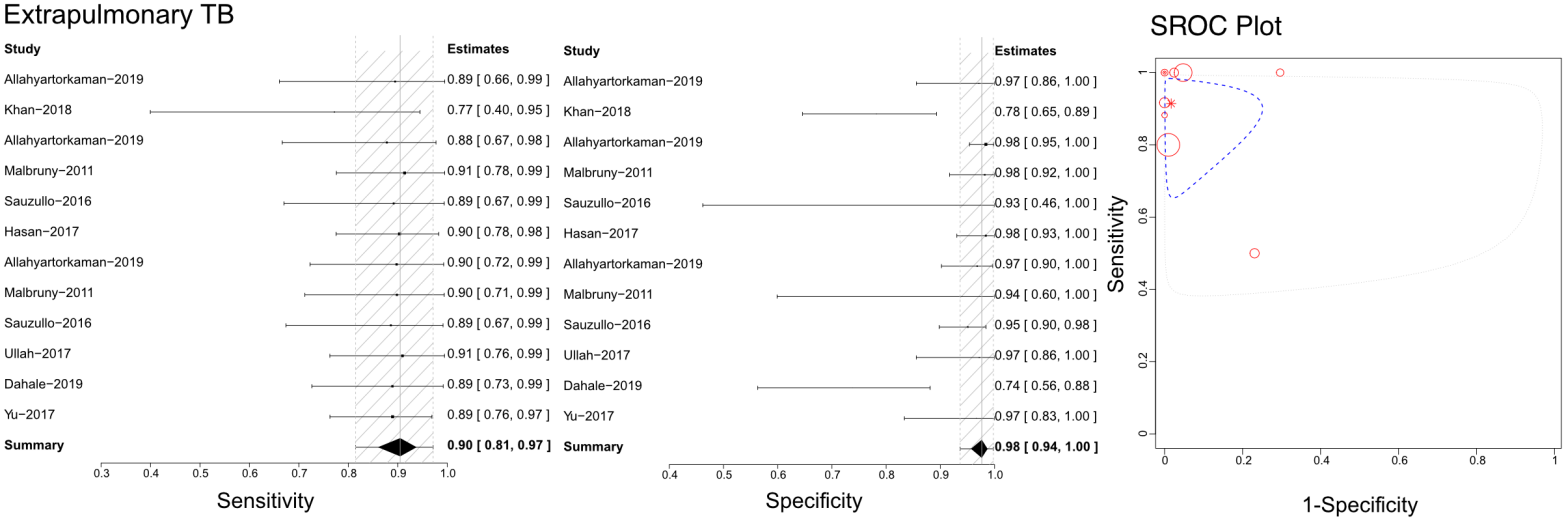
The sensitivity, specificity and SROC curve of different samples for diagnosing extrapulmonary TB based Xpert analysis.

Based on the literature we retrieved, Xpert has been used to differentiate unclassified extrapulmonary TB using compound specimens (**Figure 7**). Sensitivities, specificities, and SROCs were computed among the analyses. Twelve articles were deemed eligible to determine the diagnostic efficacy of compound specimens in Xpert assessment of unclassified extrapulmonary TB. The sensitivity of compound specimens in Xpert assessment for identifying unclassified extrapulmonary TB was 0.90 (95% CI 0.81–0.97), while its specificity was 0.98 (95%CI 0.94–1.00). The AUC of samples in the Xpert application was 0.416 ± 0.387, and the estimated AUC was 0.876 (**Figure 7**). **Supplementary Figures 7D**, **8D** present the Crosshair and posterior density plots, respectively, for the diagnostic efficacy of compound specimens in Xpert assessment of unclassified extrapulmonary TB.

### Efficacy of Xpert in identifying other types of TB

In accordance with our pre-defined inclusion and exclusion criteria, we have identified several studies that have demonstrated the diagnostic accuracy of Xpert in identifying various forms of TB. One study reported on the diagnostic efficacy of urine in urinary TB, with a sensitivity of 0.69 and specificity of 1.00 (**Table 2**). Another study investigated the diagnostic efficacy of stool samples in intestinal TB, with a sensitivity of 0.36 and specificity of 0.75 (**Table 2**). Furthermore, we identified three articles that were eligible for calculating the diagnostic efficacy of ascitic fluid in peritoneal TB. The sensitivity of using ascitic fluid to diagnose peritoneal TB was found to be 0.65 (95% CI 0.44–0.83), while the specificity was 0.99 (95% CI 0.95–1.00). Therefore, the AUC of ascitic fluid in peritoneal TB for Xpert application was calculated to be 0.638 ± 0.367, with an estimated AUC of 0.824 (**Table 2**). Crosshair and posterior density plots are provided in **Supplementary Figure 7E** and **Supplementary Figure 8E**, respectively. Additionally, one article was eligible for determining the diagnostic efficacy of ascitic biopsy in peritoneal TB. The study reported a sensitivity of 1.00 and a specificity of 0.70 (**Table 2**).

**Table 2:** Summary of pooled results on other types of TB.

Sample for Xpet	Included researches	Sensitivity (95%CI)	Specificity (95%CI)	AUC (Mean ± SD)	Publication bias
Urine in urinary TB	1	0.69	1.00	–	–
Stool to intestinal TB	1	0.36	0.75	–	–
Ascitic fluid in peritoneal TB	3	0.65 (0.44,0.83)	0.99 (0.95, 1.00)	0.638 ± 0.367	Absent
Ascitic biopsy in peritoneal TB	1	1.00	0.70	–	–

CI, confidential intervals; SD, standard difference.

## Discussion

This meta-analysis aimed to evaluate the diagnostic efficacy of Xpert in identifying different types of TB infections based on the use of various specimens. Based on the results obtained from this meta-analysis, we aimed to provide recommendations for selecting the optimal specimen for detecting suspected TB infections. We conducted a comprehensive literature review to identify all types of TB and the specimens used in each Xpert application. We then calculated the pooled sensitivities, specificities, and SROCs for each pair of the applied specimen and suspected TB infection. The findings of this meta-analysis offer valuable insights for clinical practice. Since its introduction in 2010, Xpert has been shown to possess superior characteristics in identifying TB pathogens and has been applied to various clinical diseases, including COVID-19 (Lee and Song, 2021). Several meta-analyses have demonstrated the efficacy of Xpert in TB diagnosis. However, previous meta-analyses have mainly focused on a specific type of TB infection or the accuracy of Xpert with compound specimen applications or a specific specimen among unclassified types of TB infection (Steingart et al., 2014; Kohli et al., 2018; Ssengooba et al., 2020; Wang et al., 2020; Sharma et al., 2021). This study is the first meta-analysis to provide an integrative assessment of Xpert’s diagnostic efficacy in identifying different types of TB based on various specimens’ applications.

Based on our evaluation of pulmonary TB, all specimens used in the Xpert analysis demonstrated satisfactory specificity. However, there were significant differences in sensitivity assessments among the different specimens. Sputum and gastric juice, commonly used specimens, showed similar sensitivity values, around 0.95. In contrast, Xpert based on BALF had a lower sensitivity of 0.88 and an AUC of 0.879 ± 0.098, which was lower than that of sputum and gastric juice. Importantly, invasive sample harvesting by bronchoscopy is not as simple as obtaining sputum and gastric juice. Harvesting gastric juice is an invasive method, which precludes its use as the primary choice for Xpert analysis. Sputum would be the easiest and most convenient way to detect pulmonary TB but false negative results are expected with this sampling method. To overcome this issue, multiple sputum examinations would be required. Stool or biopsy samples obtained by bronchoscopy had unsatisfactory sensitivities, lower than 0.80. Thus, due to the excellent efficacy of Xpert on sputum and gastric juice samples, repeated Xpert examinations on sputum or gastric juice would be more efficient in achieving a precise diagnosis of pulmonary TB compared to using BALF, stool, and other samples. However, DiNardo et al. (DiNardo et al., 2015) found that 10–85% of individuals with presumed pulmonary TB cannot produce sputum, depending on age and disease status. Therefore, gastric juice could serve as an alternative to diagnostic specimens. Although stool had lower sensitivity, it had the highest specificity. Stool samples are also more accessible than BALF, gastric juice, and biopsies. Because obtaining BALF, gastric juice, and biopsy samples is invasive, these samples cannot be easily acquired from infants or young children. Additionally, young patients cannot produce enough sputum for Xpert analysis. Therefore, performing Xpert on gastric juice for infants and young children would still be a good alternation. Although stool samples have lower sensitivity, their high specificity implies high accuracy with multiple assessments, as confirmed in a prospective study by Sun et al. in 2021 (Donovan et al., 2020).

TB infection affecting the bone and joints results in significant morbidity and disability due to the damage inflicted on chondrocytes, which lack the ability to regenerate postnatally. Therefore, early bone and joint TB diagnosis is critical in improving patient outcomes. Our results indicate that Xpert effectively detects bone and joint TB using joint fluid and biopsy tissue samples, demonstrating similar diagnostic values. Furthermore, the accuracy of Xpert in identifying bone and joint TB can be enhanced by selecting appropriate tissue biopsy samples. Thus, using joint fluid for Xpert analysis should be considered the primary option. In a previous study, Zhou et al. conducted a retrospective study and found that the positive rate of the Xpert assay was significantly higher when using granulation tissue specimens compared to caseous necrotic tissue, sequestrum, and other necrotic connective tissues for bone and joint TB diagnosis (Zhou et al., 2021).

In evaluating extrapulmonary TB, combining multiple specimens appears to be more helpful than relying on a single sample. Although Shama et al. recommended urine samples for Xpert analysis in extrapulmonary TB diagnosis, our meta-analysis failed to identify any advantages of urine over stool or gastric juice. Given the uncertain nature of extrapulmonary TB, using multiple specimens for Xpert testing would be a superior choice. TB lymphadenitis is the most common extrapulmonary manifestation of the disease and presents diagnostic and therapeutic challenges due to its resemblance to other pathologic processes and inconsistent physical and laboratory findings. Therefore, diagnosis of TB lymphadenitis often requires lymph node biopsy (Mohapatra and Janmeja, 2009). Our analysis confirmed that Xpert analysis of biopsy samples is viable for TB lymphadenitis diagnosis. In recent years, fine-needle aspiration cytology has become a more straightforward outpatient diagnostic procedure that has replaced complete excisional node biopsy and significantly improved diagnostic accuracy (Cataño and Robledo, 2016). However, lymph node biopsy is an invasive approach and its use necessitates a risk evaluation for the recipients. Therefore, alternative specimen options should be considered. A recent study suggested that lymphoglandular fluid could be a promising alternative to conventional biopsy for Xpert analysis. Nonetheless, more studies are required to enhance the evidence and evaluate the accuracy of this approach.

The diagnostic performance of Xpert for TB and tuberculous meningitis was inferior to that for pulmonary TB, as well as bone and joint TB, but both maintained a remarkably high specificity. A meta-analysis by Kohli et al. (Kohli et al., 2018) demonstrated that Xpert achieved a specificity higher than 98% for diagnosing peritoneal TB based on compound samples. In contrast, a retrospective study by Nguyen et al. (Mai and Thwaites, 2017) indicated that Xpert played a crucial role in diagnosing tuberculous meningitis but showed poor sensitivity, which limited its ability to exclude the disease. Nevertheless, our study also confirmed the high specificity of Xpert for these diseases. Given the characteristics of these types of TB, significant improvement of sensitivity remains challenging. Repeating Xpert tests multiple times could enhance its overall sensitivity.

Limitations of this meta-analysis include: 1) possible variations in Xpert policies among different countries that could impact test results, but this factor could not be analyzed. 2) Inclusion of studies involving children or HIV-positive patients expanded our findings’ generalizability, as the aim of this study was to explore the diagnostic utility of various specimens in detecting different types of TB infection. However, this issue may affect the results, and it still needs to be considered as a limitation. 3) The meta4diag program package in R software, which was used for the analysis, did not include a heterogeneity test. Therefore, additional studies with more convincing results may be needed for potential influence factor analysis of some studies.

## Conclusion

In conclusion, Xpert has shown satisfactory diagnostic accuracy in most cases of TB infections. Nevertheless, the efficacy of detection varied depending on the specimens used for Xpert analysis. Unselected specimens for Xpert could diminish its ability to differentiate TB. Therefore, we have highlighted the critical role of specimen selection in TB detection using Xpert.

## Data availability statement

The datasets presented in this study can be found in online repositories. The names of the repository/repositories and accession number(s) can be found in the article/**Supplementary Material**.

## Author contributions

XG, YRH, YMH, KZ, and YL collected the data. XG and YRH reviewed the literature and contributed to manuscript drafting; XG and YL conceptualized and designed the study, coordinated and supervised data collection, and critically reviewed the manuscript for important intellectual content. YL were responsible for the revision of the manuscript for important intellectual content. All authors contributed to the article and approved the submitted version.

## Supplementary material

Supplementary Table 1 Essential information of included studies.

Supplementary Table 2 Traffic light plot (A) for the assessment of potential risk of bias summary, which have 5 columns (D1 for Patient selection, D2 for Index test, D3 for Reference standard, D4 for Flow and time, and 1 for overall judgements, in that order).

Supplementary Figure 1 PRISMA flow chart on citations selection.

Supplementary Figure 2 Quality assessment of the included articles. QUADAS, Quality Assessment of Diagnostic Accuracy Studies.

Supplementary Figure 3 Crosshair plots for pooled results on pulmonary TB diagnosis using Xpert.

Supplementary Figure 4 Posterior density plots for pooled results on pulmonary TB diagnosis using Xpert.

Supplementary Figure 5 Crosshair plots for pooled results on bone and joint TB diagnosis using Xpert.

Supplementary Figure 6 Posterior density plots for pooled results on bone and joint TB diagnosis using Xpert.

Supplementary Figure 7 Crosshair plots for pooled results on various TB diagnosis using Xpert. **(A)** Biopsy sample for tuberculous lymphadenitis diagnosis based on Xpert. **(B)** Cerebrospinal fluid sample for tuberculosis meningitis diagnosis based on Xpert. **(C)** Fluid sample for pleural TB diagnosis based on Xpert. **(D)** Extrapulmonary TB diagnosis based on Xpert. **(E)** Ascitic fluid for peritoneal TB diagnosis based on Xpert.

Supplementary Figure 8 Posterior density plots for pooled results on various TB diagnosis using Xpert. **(A)** Biopsy sample for tuberculous lymphadenitis diagnosis based on Xpert. **(B)** Cerebrospinal fluid sample for tuberculosis meningitis diagnosis based on Xpert. **(C)** Fluid sample for pleural TB diagnosis based on Xpert. **(D)** Extrapulmonary TB diagnosis based on Xpert. **(E)** Ascitic fluid for peritoneal TB diagnosis based on Xpert.
