# Supplementary figures and images for: Efficacy of Xpert in tuberculosis diagnosis based on various specimens: a systematic review and meta-analysis
Source: Front Cell Infect Microbiol. 2023 May 2;13:1149741. doi: 10.3389/fcimb.2023.1149741 (PMC10185844; doi:10.3389/fcimb.2023.1149741)

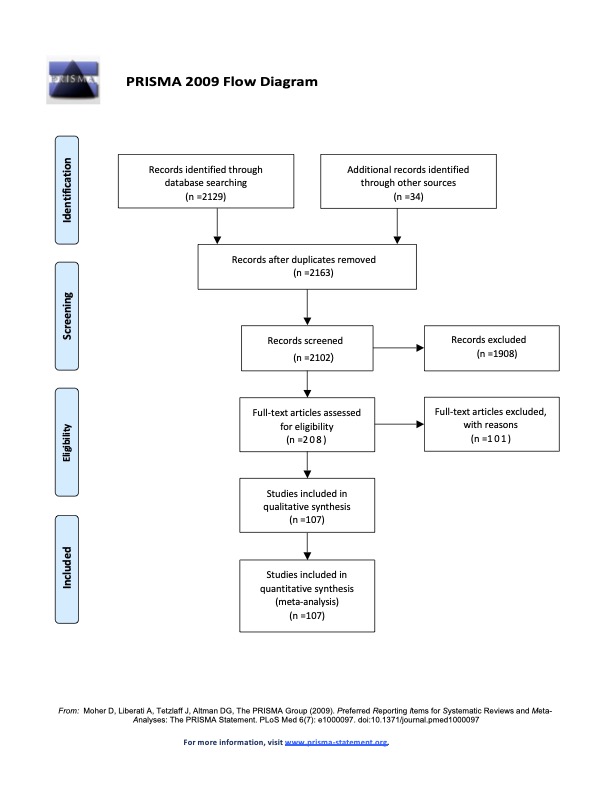

Supplement: Supplementary Figure 1 — PRISMA flow chart on citations selection. [file Image_1.jpeg]

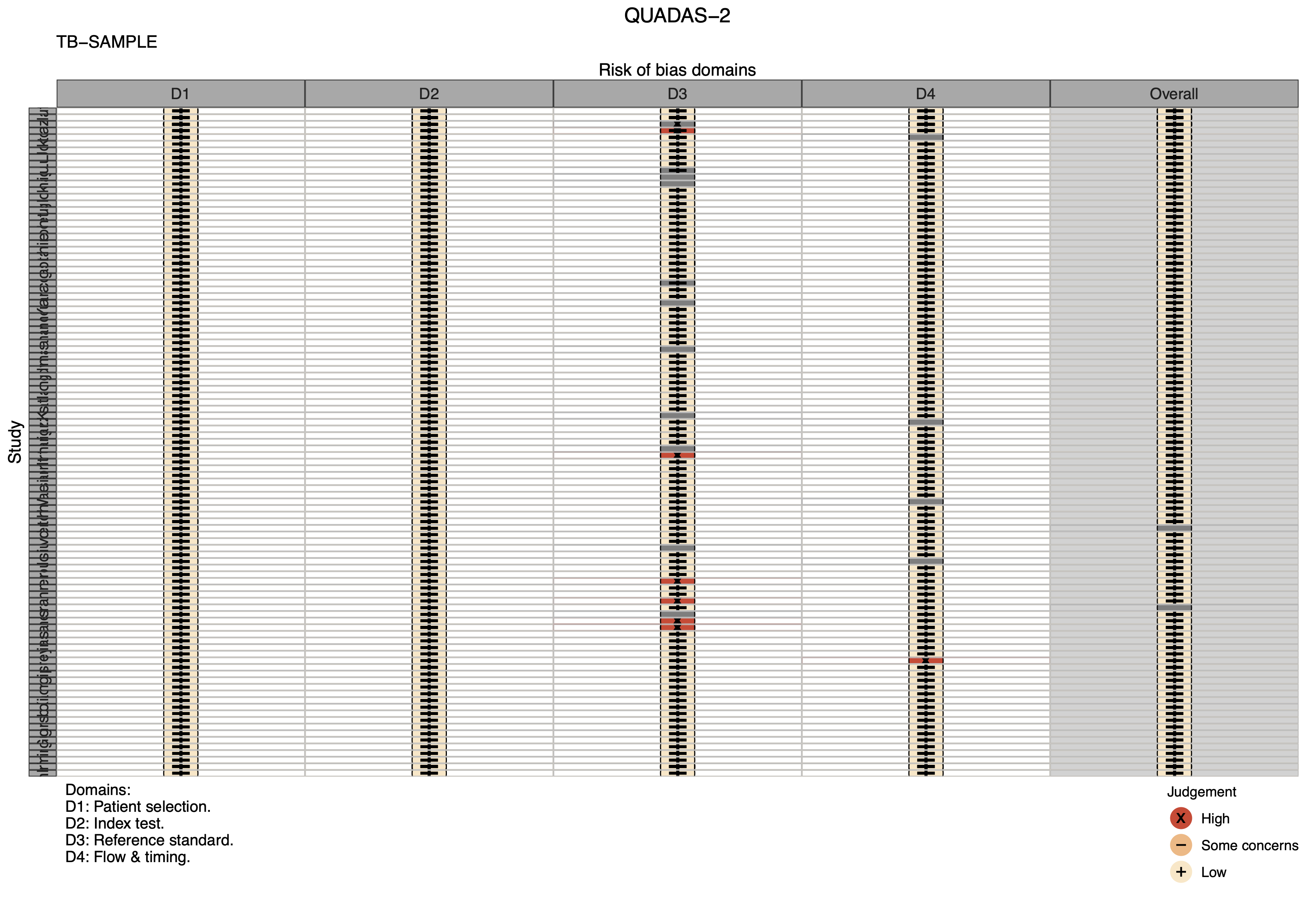

Supplement: Supplementary Figure 2 — Quality assessment of the included articles. QUADAS, Quality Assessment of Diagnostic Accuracy Studies. [file Image_2.tif]

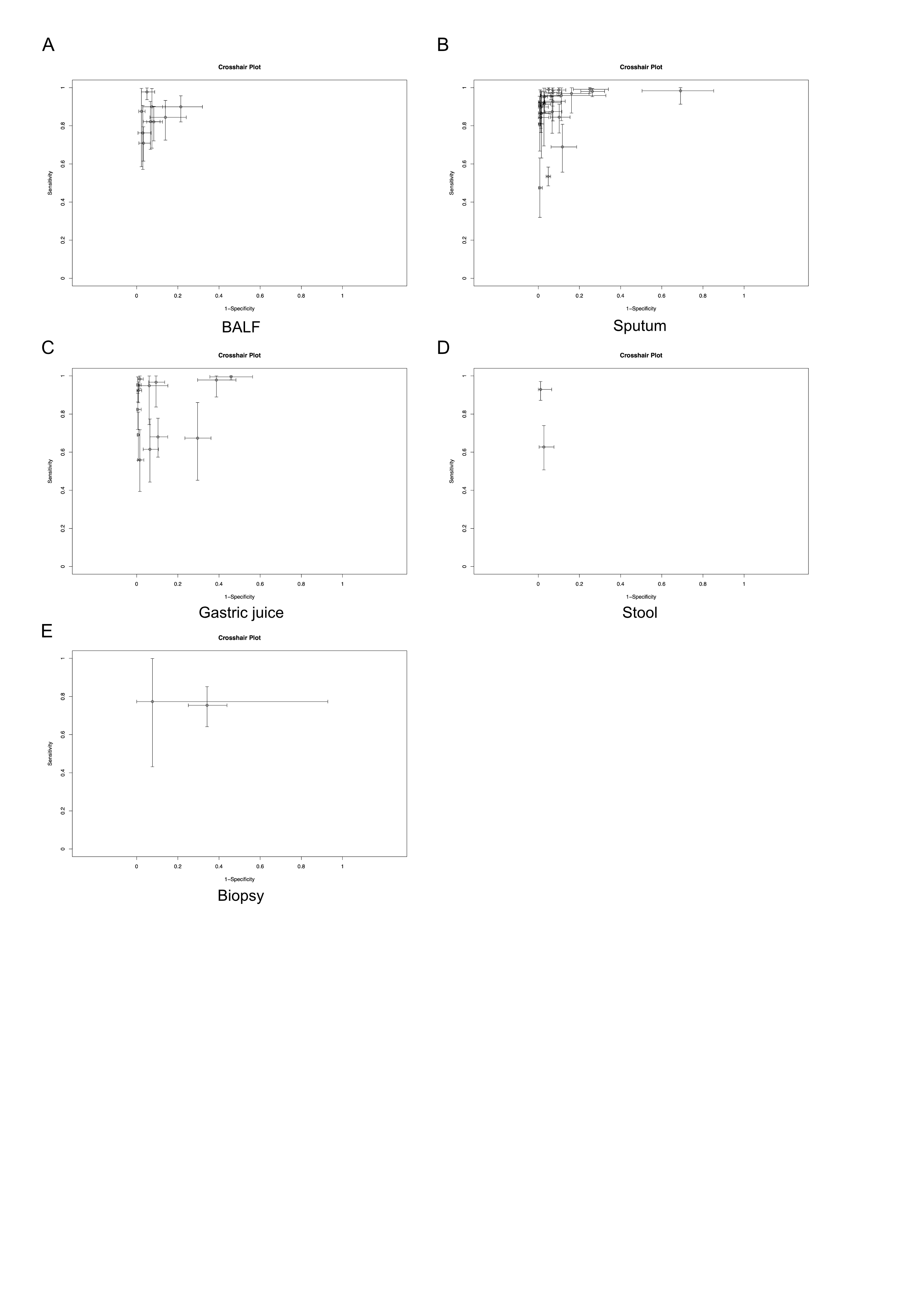

Supplement: Supplementary Figure 3 — Crosshair plots for pooled results on pulmonary TB diagnosis using Xpert. [file Image_3.tiff]

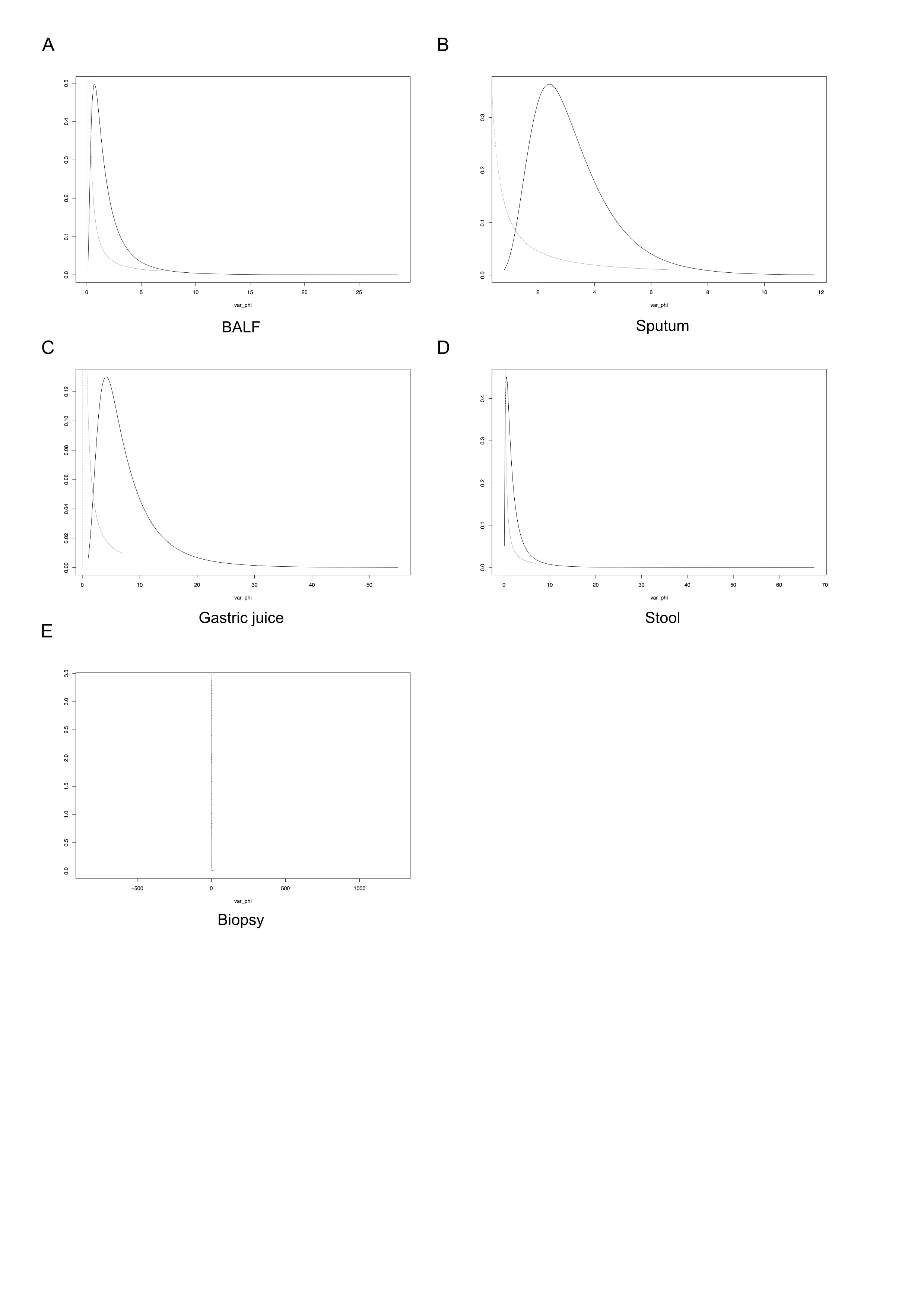

Supplement: Supplementary Figure 4 — Posterior density plots for pooled results on pulmonary TB diagnosis using Xpert. [file Image_4.tiff]

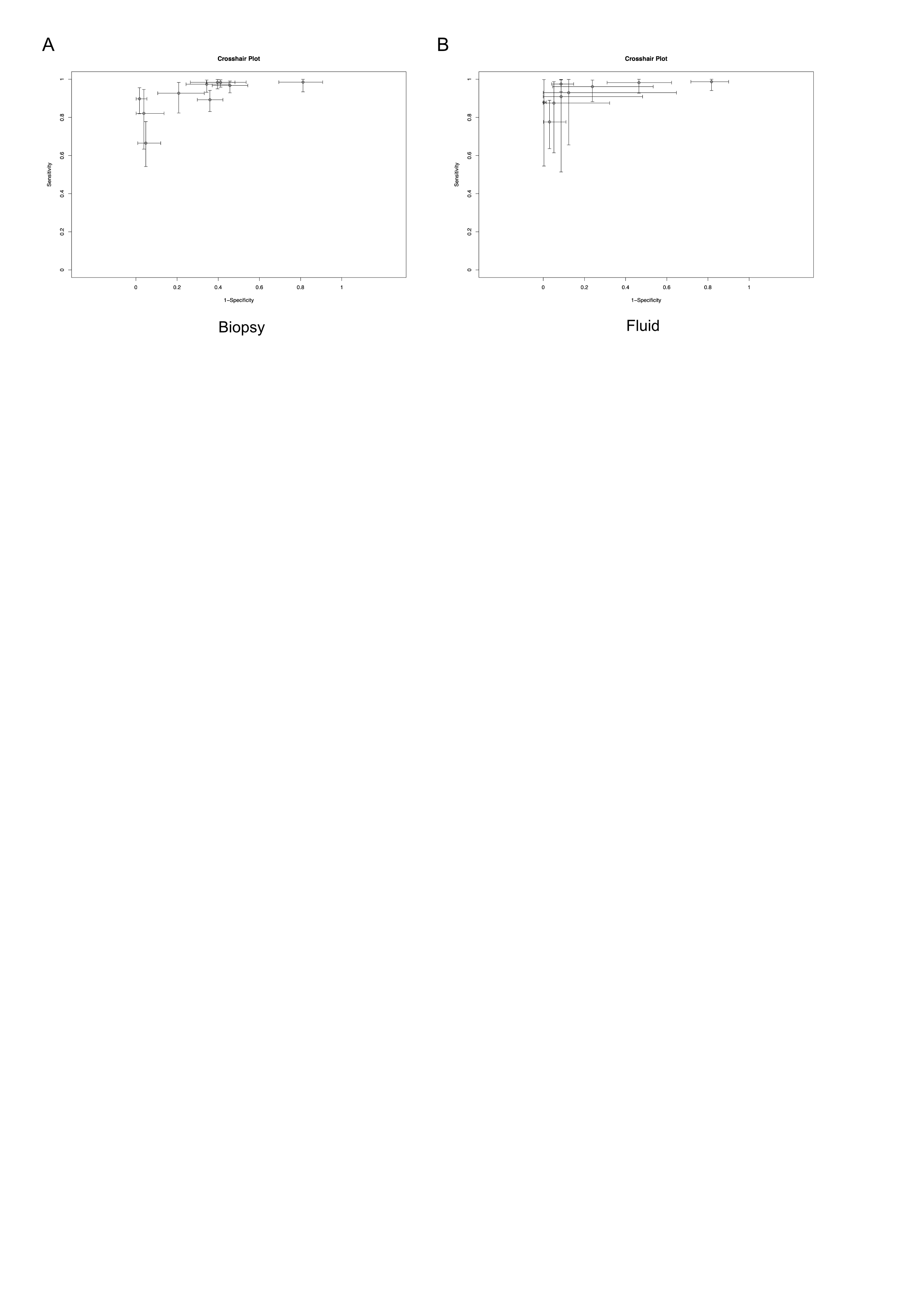

Supplement: Supplementary Figure 5 — Crosshair plots for pooled results on bone and joint TB diagnosis using Xpert. [file Image_5.tiff]

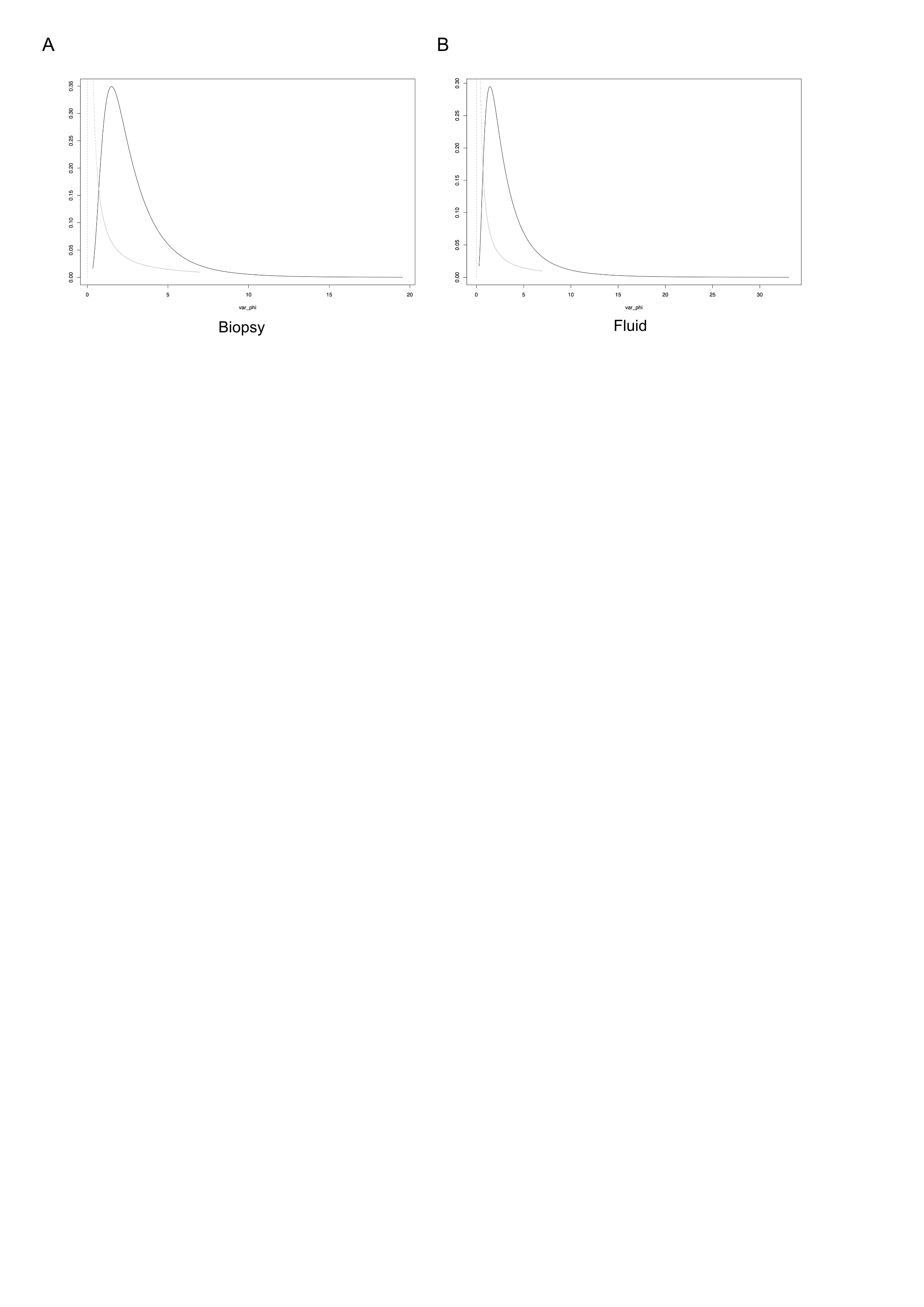

Supplement: Supplementary Figure 6 — Posterior density plots for pooled results on bone and joint TB diagnosis using Xpert. [file Image_6.tiff]

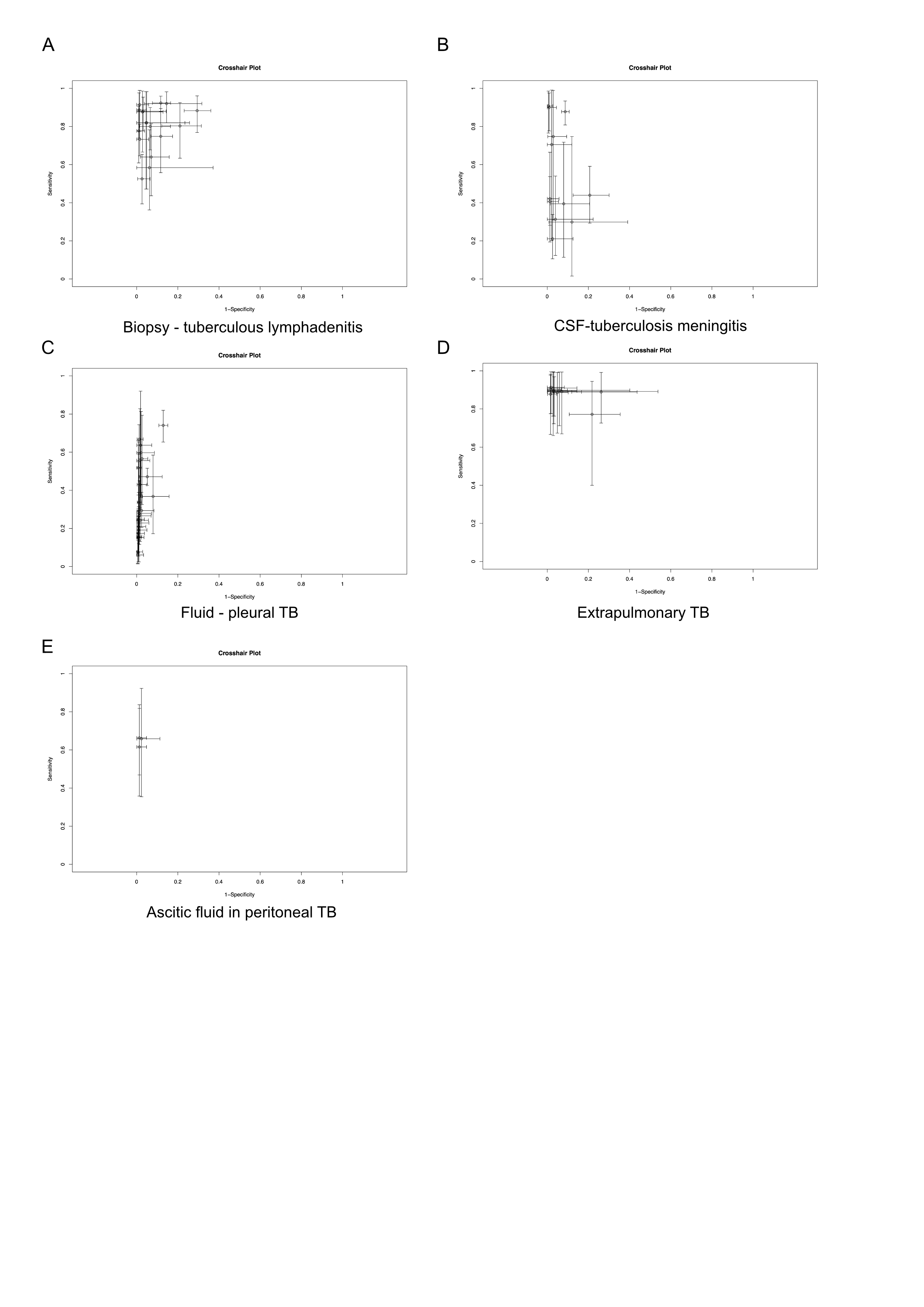

Supplement: Supplementary Figure 7 — Crosshair plots for pooled results on various TB diagnosis using Xpert. (A) Biopsy sample for tuberculous lymphadenitis diagnosis based on Xpert. (B) Cerebrospinal fluid sample for tuberculosis meningitis diagnosis based on Xpert. (C) Fluid sample for pleural TB diagnosis based on Xpert. (D) Extrapulmonary TB diagnosis based on Xpert. (E) Ascitic fluid for peritoneal TB diagnosis based on Xpert. [file Image_7.tiff]

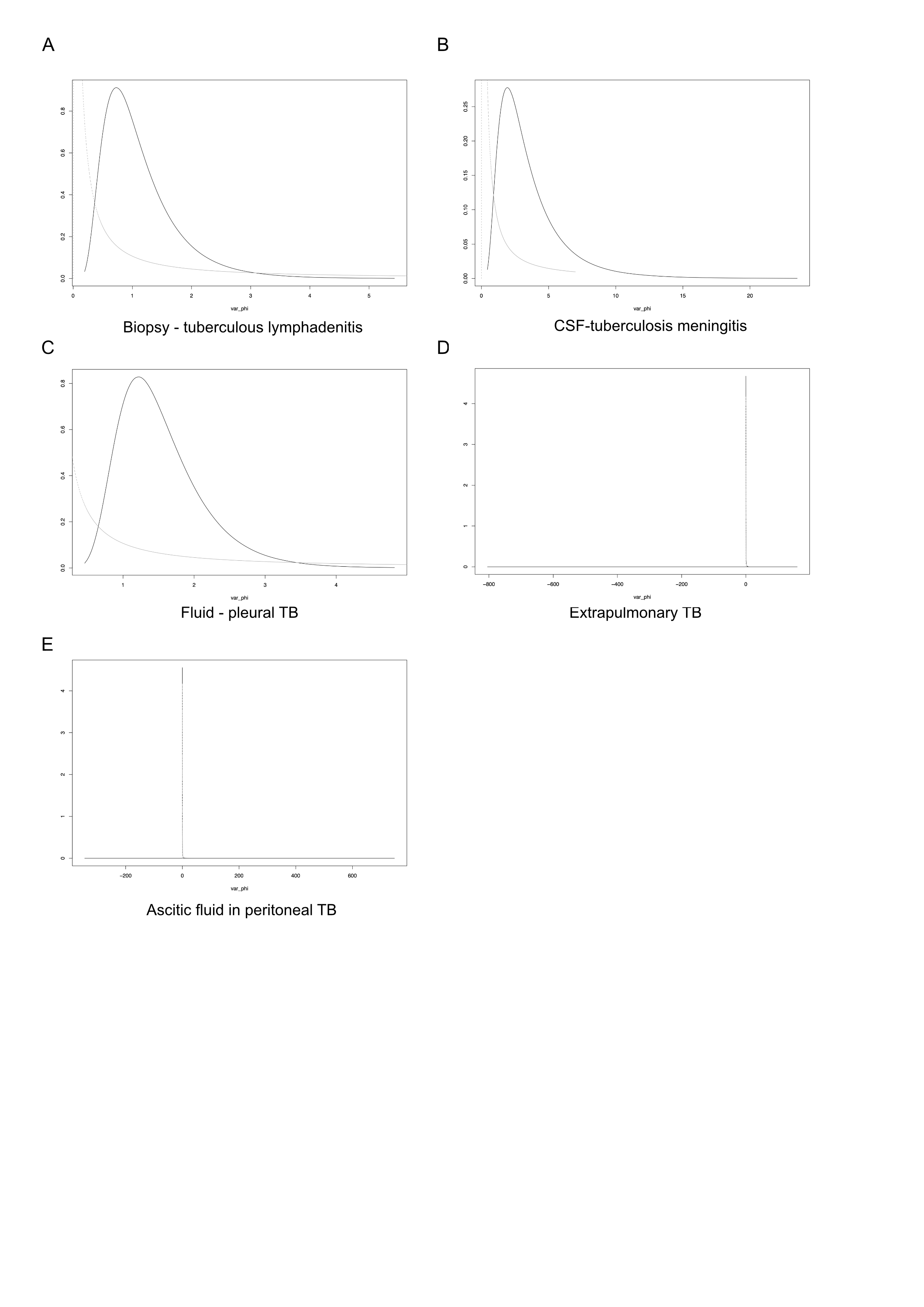

Supplement: Supplementary Figure 8 — Posterior density plots for pooled results on various TB diagnosis using Xpert. (A) Biopsy sample for tuberculous lymphadenitis diagnosis based on Xpert. (B) Cerebrospinal fluid sample for tuberculosis meningitis diagnosis based on Xpert. (C) Fluid sample for pleural TB diagnosis based on Xpert. (D) Extrapulmonary TB diagnosis based on Xpert. (E) Ascitic fluid for peritoneal TB diagnosis based on Xpert. [file Image_8.tiff]
